# COVID-19 as a putative trigger of anti-MDA5-associated dermatomyositis with acute respiratory distress syndrome (ARDS) requiring lung transplantation, a case report

**DOI:** 10.1186/s41927-022-00271-1

**Published:** 2022-07-13

**Authors:** Karolina Anderle, Klaus Machold, Hans P. Kiener, Daniel Bormann, Konrad Hoetzenecker, Silvana Geleff, Helmut Prosch, Franco Laccone, Peter M. Heil, Peter Petzelbauer, Daniel Aletaha, Stephan Blüml, Kastriot Kastrati

**Affiliations:** 1 Division of Rheumatology, Department of Medicine III, Medical University of Vienna, Waehringer Guertel 18-20, 1090 Vienna, Austria; 2 Department of Clinical Pharmacology, Medical University of Vienna, Waehringer Guertel 18-20, 1090 Vienna, Austria; 3 Department of Thoracic Surgery, Medical University of Vienna, Waehringer Guertel 18-20, 1090 Vienna, Austria; 4 Clinical Institute of Pathology, Medical University of Vienna, Waehringer Guertel 18-20, 1090 Vienna, Austria; 5 Department of Biomedical Imaging and Image-Guided Therapy, Medical University of Vienna, Waehringer Guertel 18-20, 109, Vienna, Austria; 6 Institute of Medical Genetics, Medical University of Vienna, Waehringer Guertel 18-20, 1090 Vienna, Austria; 7 Department of Dermatology, Medical University of Vienna, Waehringer Guertel 18-20, 1090 Vienna, Austria

**Keywords:** Dermatomyositis, Anti-MDA5, ARDS, Lung transplantation, COVID-19, Case report

## Abstract

**Background:**

Autoimmune disease following COVID-19 has been studied intensely since the beginning of the pandemic. Growing evidence indicates that SARS-CoV-2 infection, by virtue of molecular mimicry can lead to an antigen-mediated cross-reaction promoting the development of a plethora of autoimmune spectrum diseases involving lungs and extrapulmonary tissues alike. In both COVID-19 and autoimmune disease, the immune self-tolerance breaks, leading to an overreaction of the immune system with production of a variety of autoantibodies, sharing similarities in clinical manifestation, laboratory, imaging, and pathology findings. Anti-Melanoma Differentiation-Associated gene 5 dermatomyositis (anti-MDA5 DM) comprises a rare subtype of systemic inflammatory myopathies associated with characteristic cutaneous features and life-threatening rapidly progressive interstitial lung disease (RP-ILD). The production of anti-MDA5 autoantibodies was proposed to be triggered by viral infections.

**Case presentation:**

A 20-year-old male patient with polyarthritis, fatigue and exertional dyspnea was referred to our department. An elevated anti-MDA5 autoantibody titer, myositis on MRI, ground glass opacifications on lung CT and histological features of Wong-type dermatomyositis were confirmed, suggesting the diagnosis of an anti-MDA5 DM. Amid further diagnostic procedures, a serologic proof of a recent SARS-CoV-2 infection emerged. Subsequently, the patient deteriorated into a fulminant respiratory failure and an urgent lung transplantation was performed, leading to remission ever since (i.e. 12 months as of now).

**Conclusions:**

We report a unique case of a patient with a new-onset anti-MDA5 DM with fulminant ARDS emerging in a post-infectious stage of COVID-19, who underwent a successful lung transplantation and achieved remission. Given the high mortality of anti-MDA5 DM associated RP-ILD, we would like to highlight that the timely recognition of this condition and urgent therapy initiation are of utmost importance.

**Supplementary Information:**

The online version contains supplementary material available at 10.1186/s41927-022-00271-1.

## Background

Autoimmune phenomena following COVID-19 have been studied intensely over the last months, comprising of over 3,000 cases and more than 70 different systemic and organ-specific immune-related disorders [1], such as systemic lupus erythematosus, antiphospholipid-like syndrome, Guillain–Barré syndrome, Löfgren`s syndrome and others. The overreactive immune response and SARS-CoV-2 as putative culprit of cross-reactivity and immune-tolerance breach via molecular mimicry has been discussed broadly [2, 3].

The striking similarities between autoimmune disease and post-COVID-19 sequelae are posing a challenge for many clinicians, especially in patients presenting with unspecific cardinal, musculoskeletal, respiratory, and cutaneous symptoms.

In particular, the anti-MDA5 DM associated ILD and severe COVID-19 pneumonia with ARDS are indistinguishable not only radiographically, showing bilateral ground-glass opacities with variable degree of consolidations on radiographs, but also on histopathological findings with features of DAD and organizing pneumonia.

Anti-MDA5 DM, a subset of idiopathic inflammatory myopathies (IIM) relatively recently identified, manifests with variable degrees of cardinal symptoms, arthralgias and arthritis, proximal myopathy, and RP-ILD, the latter being pivotal for poor prognosis and high early mortality [4].

In the Caucasian adult DM populations, the anti-MDA5 DM subtype accounts for approximately 7–16%, in Asian population between 11–60% of cases. The prevalence of ILD in anti-MDA5 DM patients differs strongly among populations. Whereas in Japan and East Asia the occurrence of ILD in anti-MDA5 DM patients varies between 82 to 100%, and RP-ILD from 39 to 100%, it seems to be less common in Caucasians, i.e. ILD in 38 to 73%, and RP-ILD in 20 to 57% anti-MDA5 DM patients [5].

The RP-ILD is relatively less frequent in the juvenile DM-type and more prevalent in patients with co-present anti-Ro-52 antibodies [6]. RP-ILD is afflicted with a 1-year mortality of over 50% despite combative immunosuppression, often complicated by opportunistic infections [7].

MDA5, a cytoplasmic pattern recognition receptor encoded by IFIH1 gene, is a retinoic acid-inducible gene I like receptor (RLR) involved in the innate immune response [8]. It is sensitive to viral dsRNA, including SARS-CoV-2 [9] and its stimulation activates downstream signaling including MAVS and IRF3 pathway and synthesis of type I and III interferons to restrict viral replication and to trigger immune response [10].

## Case presentation

A 20-year-old male patient of Bangladeshi origin was referred to the outpatient clinic of our rheumatology department with polyarthritis (week 0, timeline of events shown in Additional file 1: Fig. S1).

During the first visit, the patient reported polyarthralgia, fatigue and exertional dyspnea for 3–4 weeks. Weight loss, sore throat, and a non-productive cough had been present for 2 months and he had a single episode of elevated temperature one week prior. The patient was non-smoker and did not report any prior diseases, allergies or recent travels. He had moved to Austria 3 years prior and none of the household members shared any similar symptoms at any point in time.

**Fig. 1:**
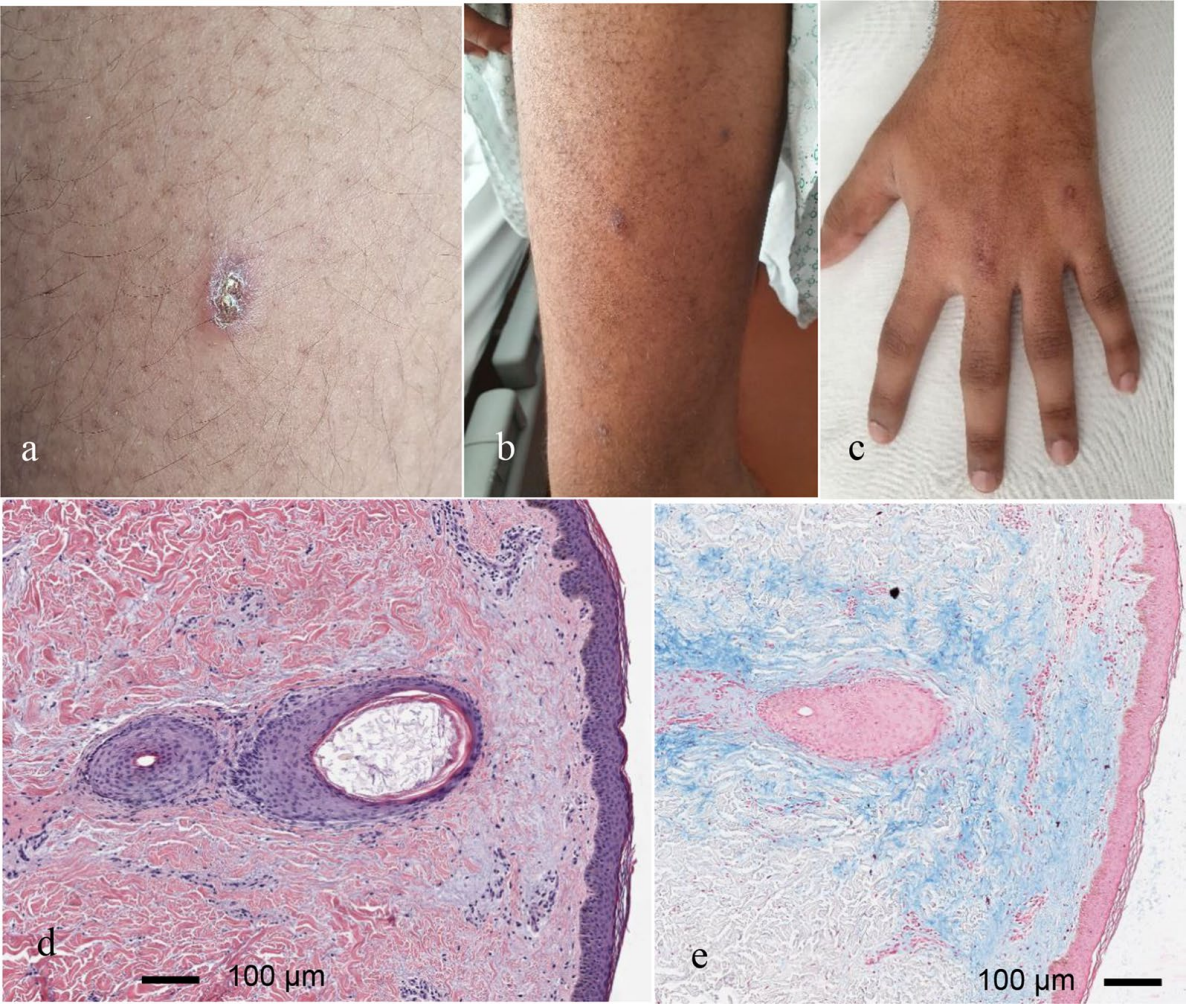
**a**–**c** Images of patient’s skin lesions at week 3: small keratotic papules, hyperpigmentation over the PIP and DIP joints, Gottron`s sign; **d** Microscopic examination of H&E stained sections: keratotic plugs within dilated follicular ostia similar to that seen in keratosis pilaris and a sparse perivascular lymphocytic infiltrate; **e** Marked perifollicular deposition of mucin on alcian blue staining. The slides were digitalized with ScanScope CS2 (Aperio Technologies, Vista, CA), no further image processing was performed

On clinical examination, the proximal interphalangeal (PIP), metacarpophalangeal (MCP) and carpo-metacarpal (CMC) joints were swollen and tender, subtle proximal muscular weakness in the lower extremities was noted. The skin over the PIP and DIP joints showed minimal non-palpable brownish discoloration, shoulders and upper arms were covered with small hyperkeratotic papules, Gottron`s sign was present (shown in Fig. 1a–c).

Laboratory investigations showed slightly elevated C-reactive protein (CRP) 0.6 mg/dL, ferritin 3933 μg/L, aspartate aminotransferase (ASAT) 262 U/L, alanine aminotransferase (ALAT) 240 U/L, gamma-glutamyl transferase (GGT) 197 U/L, lactate dehydrogenase (LDH) 575 U/L, creatine kinase (CK) 246 U/L, and aldolase 20.8 U/L.

Positive IgG antibodies (Ab) without IgM Ab against cytomegalo-, Epstein-Barr-, herpes simplex 1 and 2 and varicella-zoster-virus were detected. An interferon-releasing assay with mycobacterium tuberculosis ESAT-6 and CFP-10 antigens was non-reactive.

Immuno-serology revealed positive anti-nuclear antibodies (ANA) with fine speckled pattern at a titer of 1:320, anti-Ro-60 Ab at 23 U/mL (ULN ≤ 10 U/mL) and, notably anti-MDA-5 Ab at 14 U/mL (ULN ≤ 10 U/mL).

Microscopic examination of hematoxylin–eosin (H&E) stained sections of the skin revealed keratotic plugs within dilated follicular ostia similar to that seen in keratosis pilaris (shown in Fig. 1d). In addition, there was a sparse perivascular lymphocytic infiltrate and a marked perifollicular deposition of acidic mucins as seen in the alcian blue staining (shown in Fig. 1e).

**Fig. 2:**
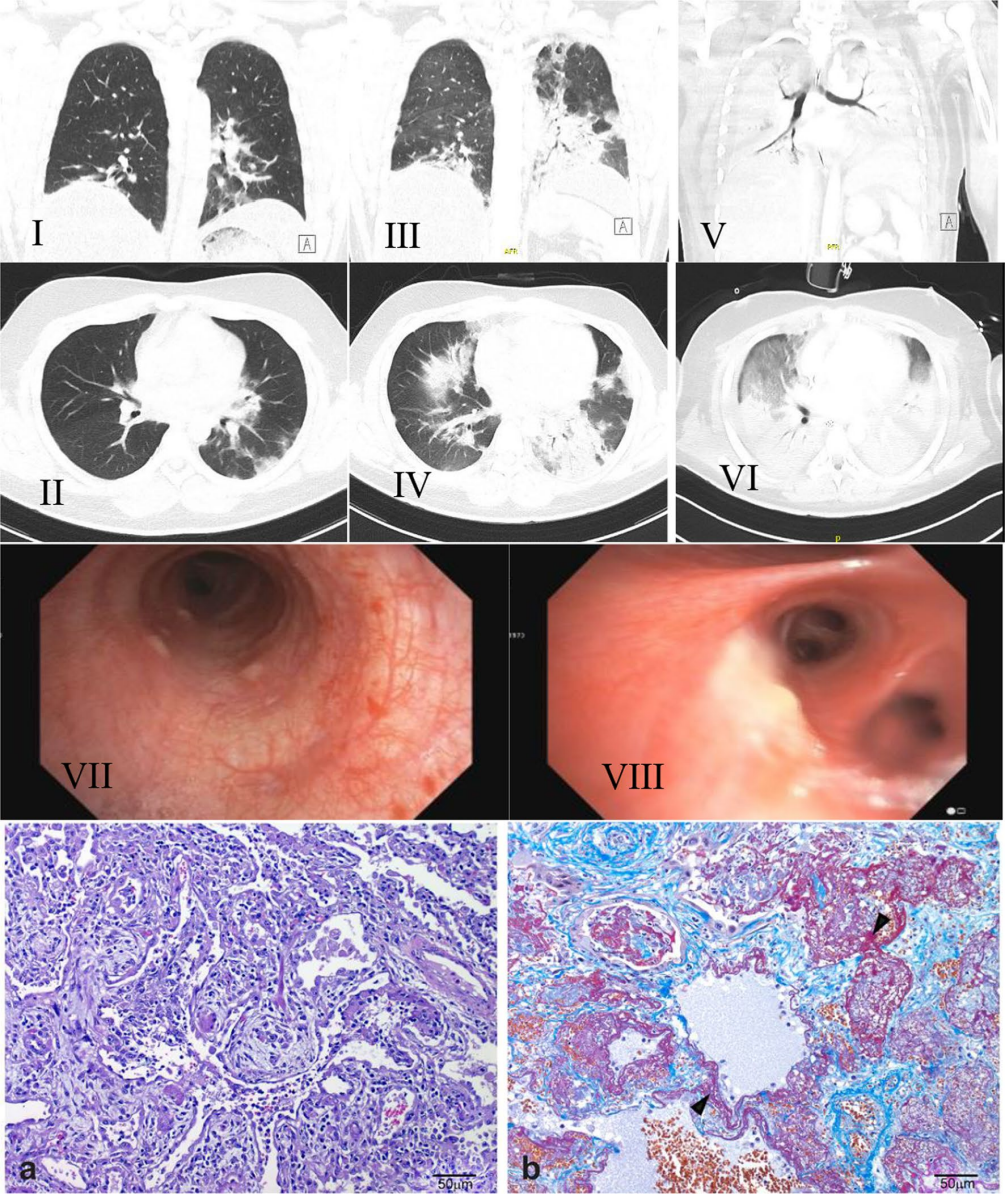
** I**, **II**: A chest CT at week 2 showed patchy ill-defined consolidations and areas of ground glass opacifications in the periphery of both lower lobes, mostly pronounced in the left lower lobe and subtle thickening of the bronchial walls;** III**,** IV**: Progression of the ground glass opacities and consolidations in both lower lobes as well as mediastinal lymphadenopathy on chest CT at week 6;** V**,** VI**: Chest CT at week 8: extensive diffuse opacification of both lungs with a ventro-dorsal density gradient with ground glass opacities in the non-dependent regions of the lungs and consolidations in the dependent regions, compatible with a diffuse alveolar damage. There was no evidence of pneumomediastinum. Clinically, the patient fulfilled criteria of an ARDS and was on ECMO;** VII**: Fibreoptic bronchoscopy showing multiple white nodular plaques, spanning from the larynx throughout most of the bronchial tract at week 4;** VIII**: Significant progression of organizing pneumonia with DAD, diffuse leukoplakia and acute bronchitis with complete squamous metaplasia at week 6;** a**,** b**: Histopathology of the lung-explant (H&E staining × 400): Acute lung damage with focal signs of organization;** a**: Features of organizing DAD and organizing pneumonia;** b**: Trichrome stain shows alveolar fibrin deposits (so-called hyaline membranes, arrowheads). The lung histopathology slides were observed in an Olympus BX46 microscope with a Pan XApo 10 × objective (final magnification 400x). Representative areas were selected, and images taken with a ProgRes C5 camera (Jenaoptik) and the corresponding ProgRes MacCapture Pro Application (2013). Except for illumination adjustment (Autofocus mode) no further enhancements of pictures were deemed necessary

Computed tomography (CT) of the thorax (shown in Fig. 2I, II) at week 2 showed patchy ill-defined consolidations and areas of ground glass opacifications in the periphery of both lower lobes and subtle thickening of the bronchial walls and hepatic steatosis.

Based on the respiratory and musculoskeletal symptoms, skin changes clinically and histologically compatible with dermatomyositis, laboratory, and auto Ab profile, anti-MDA5 associated hypomyopathic dermatomyositis with interstitial lung disease (DM-ILD) was suspected.

The patient was admitted to our ward for bronchoscopy with bronchoalveolar lavage (BAL), muscle biopsy, and subsequent initiation of immunosuppressive therapy. A negative rt-PCR test for SARS-CoV-2 was obtained on admission.

**Fig. 3:**
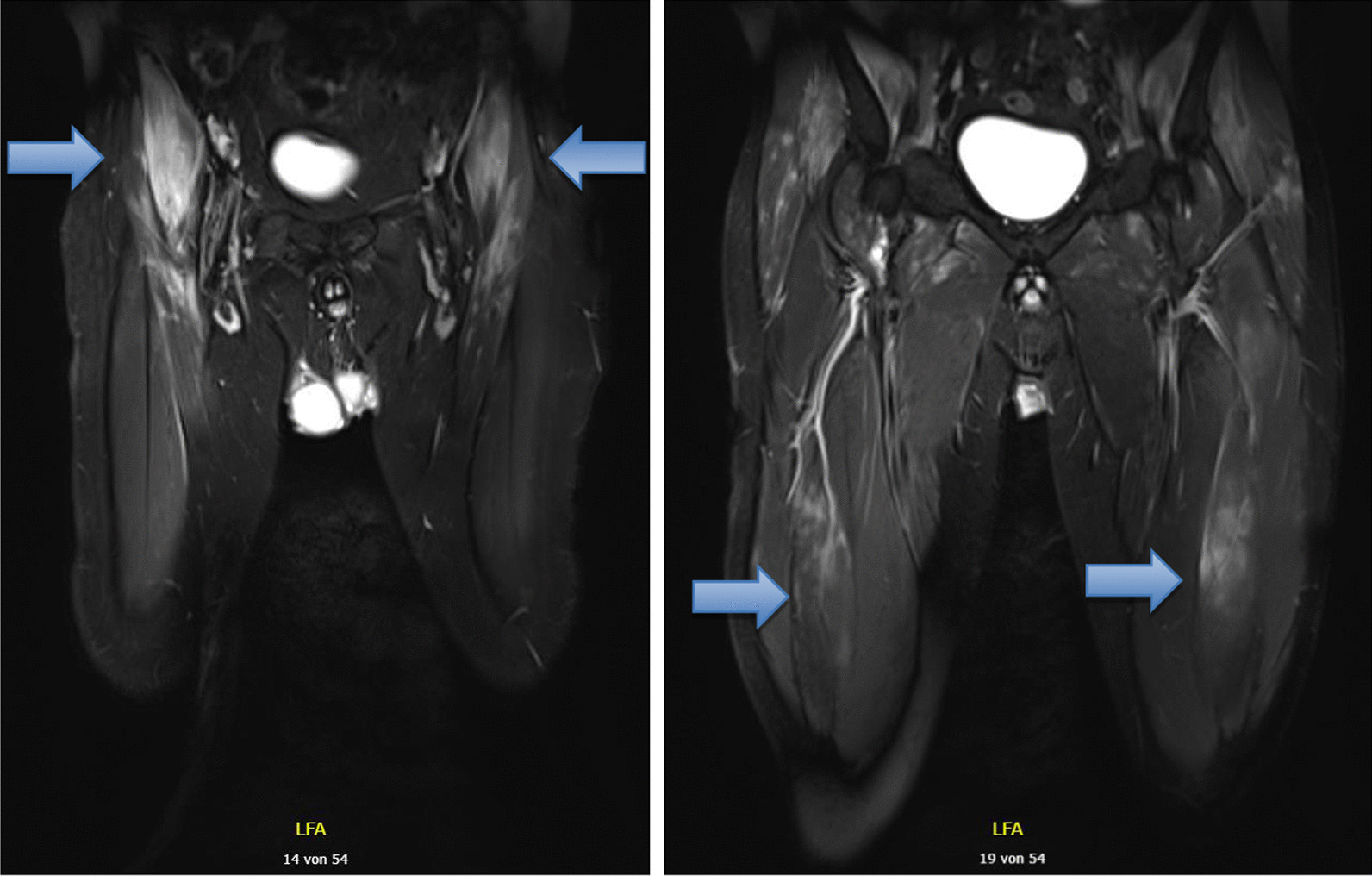
3-Tesla, gadolinium contrast enhanced MRI, T2 sequence at week 3 revealed bilateral signal enhancement in tensor fasciae latae, sartorius muscles, gluteus minimus, iliopsoas muscles (blue arrows) and other thigh muscles, with maximal involvement of the right iliopsoas muscle. The image also shows increased number and borderline size of the (reactive) inguinal lymph nodes bilaterally

A 3-Tesla, gadolinium contrast enhanced MRI revealed T2 fat saturated bilateral hyperintense signal alterations of bilateral proximal thigh muscles compatible with myositis (shown in Fig. 3).

Meanwhile, the patient`s general condition and initial symptoms, including exertional dyspnea, cough and muscle pain had improved spontaneously.

A fiberoptic bronchoscopy at week 4 showed multiple white nodular plaques, spanning from the larynx throughout most of the bronchial tract (shown in Fig. 2VII). Biopsies of the plaque lesions showed a subtle, non-specific lympho- and granulocytic infiltrate, lavage-cytology revealed mixed cell alveolitis.

On the first day after bronchoscopy, the patient became subfebrile, and the routinely performed SARS-CoV-2 rt-PCR test was newly found positive, with a cycle threshold of 41.6, and 40.2 a few hours later. The SARS-CoV-2 rt-PCR from the BAL was negative. The patient was isolated under suspicion of an incipient SARS-CoV-2 infection and the muscle biopsy was postponed.

Cycle threshold never declined below 40 and all SARS-CoV-2 tests over the following 3 days were negative. Therefore, analysis of IgG Ab against SARS-CoV-2 spike protein and nucleocapsid antigens was performed from a stored biobank sample from a timepoint where no PCR-positivity was yet detected. Both tested antibodies were positive, suggestive of a past COVID-19 infection. On further specific investigation, the patient reported, that 2.5 months prior to the presentation at our clinic, he spent several hours driving a car together with a friend, who was tested positive for SARS-CoV-2 the following day. The patient remembered elevated body temperature, cough, and general weakness after the car drive, retrospectively compatible with a mild course of COVID-19 but was never tested for SARS-CoV-2. The patient has not been vaccinated against SARS-CoV-2 at the time of presentation.

Over the next few days, the patient developed severe dyspnea with peripheral oxygen saturations below 85% and required intensified oxygen therapy. Increasing CRP and LDH levels accompanied by dry cough raised the suspicion of pneumocystis pneumonia (PCP). Trimethoprim/sulfamethoxazole was initiated before the results from testing for beta-D-Glucan, galactomannan, and PCR and cultivation for pneumocystis were available. All of these came back negative. A second diagnostic bronchoscopy was performed and showed progressive areas of leukoplakia and a highly vulnerable mucosa. There was no presence of pneumocystis or other relevant pathogens in the BAL. Lung biopsy revealed acute bronchitis with complete squamous metaplasia, and findings which were consistent with organizing pneumonia (OP), or diffuse alveolar damage (DAD), as seen in both DM-ILD and COVID-related ARDS (shown in Fig. 2VIII).

A follow-up CT of the chest at week 6 (shown in Fig. 2III, IV) revealed a progression of the ground glass opacities and consolidations in both lower lobes as well as a mediastinal lymphadenopathy.

The emergence of blisters on the lips and oropharyngeal pain raised a suspicion of a herpetiform infection. Additional microbial tests were ordered and in the light of the rapid deterioration of the patient’s general and respiratory condition, a multifaceted approach was established with glucocorticoid pulse therapy (250 mg intravenous prednisolone), acyclovir and continued trimethoprim/sulfamethoxazole. Despite this and the high-flow nasal oxygen therapy, the respiratory condition worsened, requiring transfer to intensive care unit (ICU) at week 7. The continuous respiratory deterioration led to an invasive ventilation.

CRP and IL-6-levels rose excessively (shown in Additional file 1: Fig. S2), while procalcitonin remained within normal limits, suggesting a hyperinflammatory state without relevant bacterial infection. The patient did not fulfill the criteria of a hemophagocytic lymphohistiocytosis diagnosis. The differential diagnosis of a COVID-19 relapse was also considered.

We performed a thorough literature review of therapy for RP-ILD in DM [11–13] and COVID-19 cytokine storm and associated lung injury [14–16] that was available by March 2021. After an interdisciplinary discussion, we continued high-dose glucocorticoids and started cyclophosphamide and tacrolimus because of the rapid disease progression. Colchicine was added due to the hyperinflammatory state. The administration of caspofungin, piperacillin/tazobactam and doxycycline followed for infection prophylaxis under strong immunosuppression. Intravenous immunoglobulins as well as tocilizumab and JAK inhibitors were debated. However, as already implemented aggressive immunosuppressive combination remained without desired effect and there was a serious concern about potential harm of further escalation, we did not proceed with expansion of the immunosuppression, for which we only had insufficient evidence at the time of the event. Despite high ventilation efforts, initiation of veno-venous extracorporal membrane oxygenation (ECMO) was inevitable. Considering the fulminant diffuse bilateral lung damage and the patient`s young age, listing for a highly urgent lung transplantation was the ultima ratio.

A compatible organ was allocated to the patient after a short waiting period, and the transplantation was successfully performed at week 11, after 4,5 weeks on ECMO. The primary organ function was excellent and the ECMO could be removed at the end of the operation.

Pathological examination of the H&E and SFOG trichrome stained sections of explanted organ showed extensive fibrosis with a mixed pattern of organized diffuse alveolar damage and organizing pneumonia (shown in Fig. 2a, b). Moreover, thrombotic occlusions of multiple vessels were present, which is compatible with both, a COVID-19 associated lung disease and a myositis-associated ILD [17].

Standard triple immunosuppression, including tacrolimus, mycophenolate mofetil, as well as glucocorticoids was established. The respiratory situation improved steadily, and the patient could be weaned fast. Early physiotherapy contributed to the patient`s rapid recovery and he was transferred to a rehabilitation center and further outpatient care.

One month after the lung transplantation, the antibody profile turned negative for ANA- and anti-MDA5 Ab, the anti-Ro-60 Ab were only marginally elevated (12 U/mL). On the follow-up visit, five months after the transplantation, ANA, anti-Ro Ab and anti-MDA5 Ab were negative. The patient did not report any symptoms on the follow-up visit twelve months after the lung transplantation.

Using whole exome sequencing, we investigated genetic predisposition for “myositis” phenotype according to Human Phenotype Ontology (HPO) and Online Mendelian Inheritance in Man (OMIM) database and frequency of a sequence change in the population as per findings of various databases. An extended analysis of the associated genes (shown in Additional file 1: Supplement 3) did not detect any pathogenic mutation in our patient. A pre-transplantation HLA typing was also performed.

## Discussion and conclusions

In our patient, we found multiple clinical aspects of anti-MDA5 DM. The cutaneous phenotype with Gottron`s sign, characteristic symmetric pattern of polyarthralgia and polyarthritis, proximal muscle weakness, elevated muscle enzymes, MRI positive for myositis, dermatomyositis-specific antibodies (anti-MDA5 Ab) and RP-ILD are all in line with existing evidence [5]. These multiple facets of an anti-MDA5 DM also fulfill the ENMC 2018 dermatomyositis classification criteria [18] (clinical signs: Gottron`s sign along with muscular features: proximal muscle weakness, elevated muscle enzymes and positive DM-specific Ab), which strengthened our diagnosis despite missing histopathologic confirmation due to the positive SARS-CoV-2 PCR and rapid respiratory deterioration. Hence, the added value of a muscle biopsy remains disputable, as muscle tissues of MDA5 DM patients are often unremarkable or show marginal signs of inflammation. The initially elevated anti-MDA5 Ab were undetectable after the lung transplantation and aggressive immunosuppression, which is consistent with the Ab disappearance in remission, particularly in lung transplanted patients [19, 20].

Furthermore, we do not believe the patient suffered from severe COVID-19 infection. By the time point of admission, when a negative SARS-CoV-2 rt-PCR result was obtained, the antibody titers against spike protein and nucleocapsid antigens were compatible with an infection that occurred > 14 days prior, but probably even longer, possibly as long as 10 weeks prior, when the positive contact and oligosymptomatic infection occurred. There were only 2 positive SARS-CoV-2 PCR tests from a nasal swab on the day after bronchoscopy (possibly an RNA remnant being washed out by the bronchoalveolar lavage), and all following (as well as the one at admission) rt-PCR tests for SARS-CoV-2 were negative. These 2 positive tests in a time interval of several hours had very high cycle threshold values (41.6, and 40.2), classified as non-infectious. Also, the SARS-CoV-2-PCR from the BAL was negative. In our opinion, these clues exclude the presence of active SARS-CoV-2 infection and thus a severe COVID-19.

In MDA5 DM, mucocutaneous involvement displays a whole array of phenotypes, including tender palmar papules, heliotrope rash, Gottron papules, calcinosis, mechanic's hands, hyperkeratosis, mucocutaneous ulcers, teleangiectasia, vasculitis, panniculitis, alopecia, pruritus or psoriasiform rash.

In our patient, we saw Gottron’s sign and multiple hyperkeratotic papules. Histopathological changes mimic features published under the name “Wong-type dermatomyositis”. This entity is characterized by follicular and non-follicular epidermal invaginations filled with keratin and mucin deposition within the papillary dermis. Moreover, as seen in our patient, in these cases the characteristic changes at the dermo-epithelial junctions may be missing [21, 22]. Hence, the clinicians need to be aware of the polymorphous spectrum of the cutaneous DM, which may cause diagnostic delay.

Moreover, arthralgias and non-erosive arthritis, which are seen in around 80% of anti-MDA5 DM patients, resemble rheumatoid arthritis pattern and can lead to misdiagnosis [23].

According to the emerging literature, various auto-antibodies are found in up to a half of hospitalized COVID-19 patients and they seem to be low-titer, transient and fluctuating with the disease course [5, 24, 25]. In the retrospective Chinese cohort study conducted by Wang et al. [26] with 274 adult patients between 44 and 73 years of age hospitalized with COVID-19, anti-MDA5 autoantibodies were present in 48.2% of patients and MDA5 Ab titers were significantly higher in non-survivors compared to survivors. The non-survivors were 64 years old (IQR, 54–73) and had a mean anti-MDA5 Ab titer of 5.91 U/l (IQR, 3.82–10.77), which was lower than the initial titer of 14 U/ml in our patient, measured before the acute deterioration. However, this study reports the anti-MDA5 Ab seroconversion of patients with an active COVID-19 without assessment of clinical features related to the “true” dermatomyositis. While the dysregulation of viral antigen interaction with MDA5 has been proposed in the early pathogenesis of autoimmune diseases like systemic lupus erythematosus or type 1 diabetes [27, 28], low Ab titers in absence of related clinical features should not be automatically translated into a diagnosis of autoimmune disease. This is mainly because type I interferon signaling pathway activation is a part of physiological innate immune response of cells infected with SARS-CoV-2 [29].

A few patient cases of different dermatomyositis subtypes possibly linked to a COVID-19 infection were reported throughout the pandemic, however, only half included sufficient clinical information to apply classification criteria or provided robust enough evidence, so that causality can be considered [30].

It has been discovered that rare loss-of-function variations in IFIH1 gene seem to be protective against autoimmune disease, whereas increased function of IFIH1/MDA5 is linked to a risk of autoimmune disease [27]. The extended analysis of associated genetic predispositions using whole exome sequencing did not reveal SNPs with known associations for any form of myositis in our patient, including HLA isotypes [31, 32]. Interestingly, HLA-typing showed the presence of DRB1*15:02, DRB5*01:02, DQB1*05:01 and DQB1*06:01 alleles in our patient, which are significantly enriched in anti-Scl70 + systemic sclerosis patients with progressive interstitial lung disease [33, 34].

Yet the exact molecular mechanisms and individual susceptibility in the development of an autoimmune disease after COVID-19 merit further investigation.

Taken together, it is highly likely that in this young and otherwise healthy patient, an MDA5 positive DM with RP-ILD was triggered by SARS-CoV-2 infection. Aside from the known pathophysiological mechanisms of viral MDA5 Ab induction, the latency of several weeks in the postinfectious symptom onset corresponds well with the literature of post-COVID autoimmune phenomena [1].

Reports on lung transplantation and its outcome in anti-MDA5 DM patients are scarce, especially in young patients. Given the mortality rate of almost 100% for patients with anti-MDA5 RP-ILD requiring ECMO, the timely recognition of this condition and urgent action are of utmost importance.

To our knowledge, this is the first case of a successful high-urgency lung transplantation in COVID-19-triggered dermatomyositis with RP-ILD.

## Supplementary Information

**Additional file 1**. Supplementary figures.

## Data Availability

All data generated or analyzed during this study are included in this article or its supplementary material file. Further enquiries can be directed to the corresponding author.

## Abbreviations

Ab: Antibody
ASAT: Aspartate aminotransferase
ALAT: Alanine aminotransferase
ANA: Antinuclear antibodies
BAL: Bronchoalveolar lavage
CFP-10: 10-kDa culture filtrate protein
COVID-19: Coronavirus disease 2019
CK: Creatine kinase
CMC: Carpometacarpal
CRP: C-reactive protein
CT: Computed tomography
DAD: Diffuse alveolar damage
DIP: Distal interphalangeal
DM: Dermatomyositis
dsRNA: Double-stranded ribonucleic acid
ECMO: Extracorporeal membrane oxygenation
ESAT-6: 6-kDa early secreted antigenic target
GGT: Gamma-glutamyl transferase
H&E: Hematoxylin and eosin
HLA: Human leukocyte antigen
HPO: Human phenotype ontology
ICU: Intensive care unit
IFIH1: Interferon-induced helicase C domain-containing protein 1
IIM: Idiopathic inflammatory myopathy
IL: Interleukin
ILD: Interstitial lung disease
IRF3: Interferon regulatory factor 3
LDH: Lactate dehydrogenase
MAVS: Mitochondrial antiviral signaling protein
MCP: Metacarpophalangeal
MDA5: Melanoma differentiation-associated protein 5
MRI: Magnetic resonance imaging
OMIM: Online Mendelian Inheritance in Man
OP: Organizing pneumonia
PCP: Pneumocystis pneumonia
PIP: Proximal interphalangeal
RP-ILD: Rapidly progressive interstitial lung disease
RLR: Retinoic acid-inducible gene I like receptor
rt-PCR: Reverse transcription polymerase chain reaction
SARS-CoV-2: Severe acute respiratory syndrome coronavirus 2
SFOG: Säurefuchsin-Orange-G, translated: acid-fuchsin, orange-G
ULN: Upper limit of normal

## References

[1] Ramos-Casals M, Brito-Zerón P, Mariette X (2021). Systemic and organ-specific immune-related manifestations of COVID-19. Nat Rev Rheumatol.

[2] Liu Y, Sawalha AH, Lu Q (2021). COVID-19 and autoimmune diseases. Curr Opin Rheumatol.

[3] Moody R, Wilson K, Flanagan KL, Jaworowski A, Plebanski M (2021). Adaptive immunity and the risk of autoreactivity in COVID-19. Int J Mol Sci.

[4] Sato S (2005). Autoantibodies to a 140-kd polypeptide, CADM-140, in Japanese patients with clinically amyopathic dermatomyositis. Arthritis Rheum.

[5] Nombel A, Fabien N, Coutant F. Dermatomyositis with anti-MDA5 antibodies: bioclinical features pathogenesis and emerging therapies. Front Immunol 2021. 10.3389/fimmu.2021.773352. 10.3389/fimmu.2021.773352 PMC8564476 34745149

[6] Sabbagh S (2019). Anti-Ro52 autoantibodies are associated with interstitial lung disease and more severe disease in patients with juvenile myositis. Ann Rheum Dis.

[7] Lian X (2020). Mortality risk prediction in amyopathic dermatomyositis associated with interstitial lung disease: the FLAIR model. Chest.

[8] Kato H (2006). Differential roles of MDA5 and RIG-I helicases in the recognition of RNA viruses. Nature.

[9] Yin X (2021). MDA5 governs the innate immune response to SARS-CoV-2 in lung epithelial cells. Cell Rep.

[10] Onomoto K, Onoguchi K, Yoneyama M (2021). Regulation of RIG-I-like receptor-mediated signaling: interaction between host and viral factors. Cell Mol Immunol.

[11] Matsuda KM (2020). Combined immunosuppressive therapy provides favorable prognosis and increased risk of cytomegalovirus reactivation in anti-melanoma differentiation-associated gene 5 antibody-positive dermatomyositis. J Dermatol.

[12] Aoyama J (2019). Anti-MDA5 antibody-positive rapidly progressive interstitial pneumonia without cutaneous manifestations. Respir Med Case Rep.

[13] Kurita T, Yasuda S, Amengual O, Atsumi T (2015). The efficacy of calcineurin inhibitors for the treatment of interstitial lung disease associated with polymyositis/dermatomyositis. Lupus.

[14] Deftereos SG (2020). Effect of colchicine vs standard care on cardiac and inflammatory biomarkers and clinical outcomes in patients hospitalized with coronavirus disease 2019: the GRECCO-19 randomized clinical trial. JAMA Netw Open.

[15] Scarsi M (2020). Association between treatment with colchicine and improved survival in a single-centre cohort of adult hospitalised patients with COVID-19 pneumonia and acute respiratory distress syndrome. Ann Rheum Dis.

[16] Puxeddu I (2021). COVID-19: the new challenge for rheumatologists. One year later. Exp Rheumatol.

[17] Kondo Y (2021). COVID-19 shares clinical features with anti-melanoma differentiation-associated protein 5 positive dermatomyositis and adult Still's disease. Clin Exp Rheumatol.

[18] Mammen AL (2020). 239th ENMC International Workshop: Classification of dermatomyositis, Amsterdam, the Netherlands, 14–16 December 2018. Neuromuscul Disord.

[19] Marchiset A (2021). High-emergency lung transplantation for interstitial lung disease associated with anti-MDA5 dermatomyositis: a case report. Transplant Proc.

[20] Muro Y, Sugiura K, Hoshino K, Akiyama M (2011). Disappearance of anti-MDA-5 autoantibodies in clinically amyopathic DM/interstitial lung disease during disease remission. Rheumatology.

[21] Mutasim DF, Egesi A, Spicknall KE (2016). Wong-type dermatomyositis: a mimic of many dermatoses. J Cutan Pathol.

[22] Wong KO. Dermatomyositis: a clinical of twenty-three cases in Hong Kong. Br J Dermatol 1969:81:544–547. 10.1111/j.1365-2133.1969.tb16031.x 10.1111/j.1365-2133.1969.tb16031.x 5794943

[23] Hall JC (2013). Anti-melanoma differentiation–associated protein 5–associated dermatomyositis: expanding the clinical spectrum. Arthritis Care Res (Hoboken).

[24] Chang SE (2021). New-onset IgG autoantibodies in hospitalized patients with COVID-19. Nat Commun.

[25] De Santis M (2021). Environmental triggers for connective tissue disease: the case of COVID-19 associated with dermatomyositis-specific autoantibodies. Curr Opin Rheumatol.

[26] Wang G, et al. Presence of anti-MDA5 antibody and its value for the clinical assessment in patients with COVID-19: a retrospective cohort study. Front Immunol 2021. 10.3389/fimmu.2021.79134 10.3389/fimmu.2021.791348 PMC8720853 34987516

[27] Oliveira L, Sinicato NA, Postal M, Appenzeller S, Niewold TB (2014). Dysregulation of antiviral helicase pathways in systemic lupus erythematosus. Front Genet.

[28] Lincez PJ, Shanina I, Horwitz MS (2015). Reduced expression of the MDA5 Gene IFIH1 prevents autoimmune diabetes. Diabetes.

[29] Sampaio NG (2021). The RNA sensor MDA5 detects SARS-CoV-2 infection. Sci Rep.

[30] Hannah JR (2022). Skeletal muscles and Covid-19: a systematic review of rhabdomyolysis and myositis in SARS-CoV-2 infection. Clin Exp Rheumatol.

[31] Chen Z (2017). HLA-DRB1 alleles as genetic risk factors for the development of anti-MDA5 antibodies in patients with dermatomyositis. J Rheumatol.

[32] Gono T (2012). Brief Report: Association of HLA–DRB1*0101/*0405 with susceptibility to anti–melanoma differentiation–associated gene 5 antibody–positive dermatomyositis in the Japanese population. Arthritis Rheum.

[33] Louthrenoo W (2013). Association of HLA-DRB1*15:02 and DRB5*01:02 allele with the susceptibility to systemic sclerosis in Thai patients. Rheumatol Int.

[34] Zhou XD (2013). Association of HLA-DQB1*0501 with scleroderma and its clinical features in Chinese population. Int J Immunopathol Pharmacol.

