# Supplementary material for: COVID-19 as a putative trigger of anti-MDA5-associated dermatomyositis with acute respiratory distress syndrome (ARDS) requiring lung transplantation, a case report
Source: BMC Rheumatol. 2022 Jul 13;6:42. doi: 10.1186/s41927-022-00271-1 (PMC9277832; doi:10.1186/s41927-022-00271-1)
Supplement: Supplementary file 1 — Additional file 1. Supplementary figures. [file 41927_2022_271_MOESM1_ESM.docx]

**COVID-19 as a putative trigger of anti-MDA5-associated dermatomyositis with acute respiratory distress syndrome (ARDS) requiring lung transplantation, A case report**

**Supplementary material**

**Fig. S1**: Event summary on a timeline axis


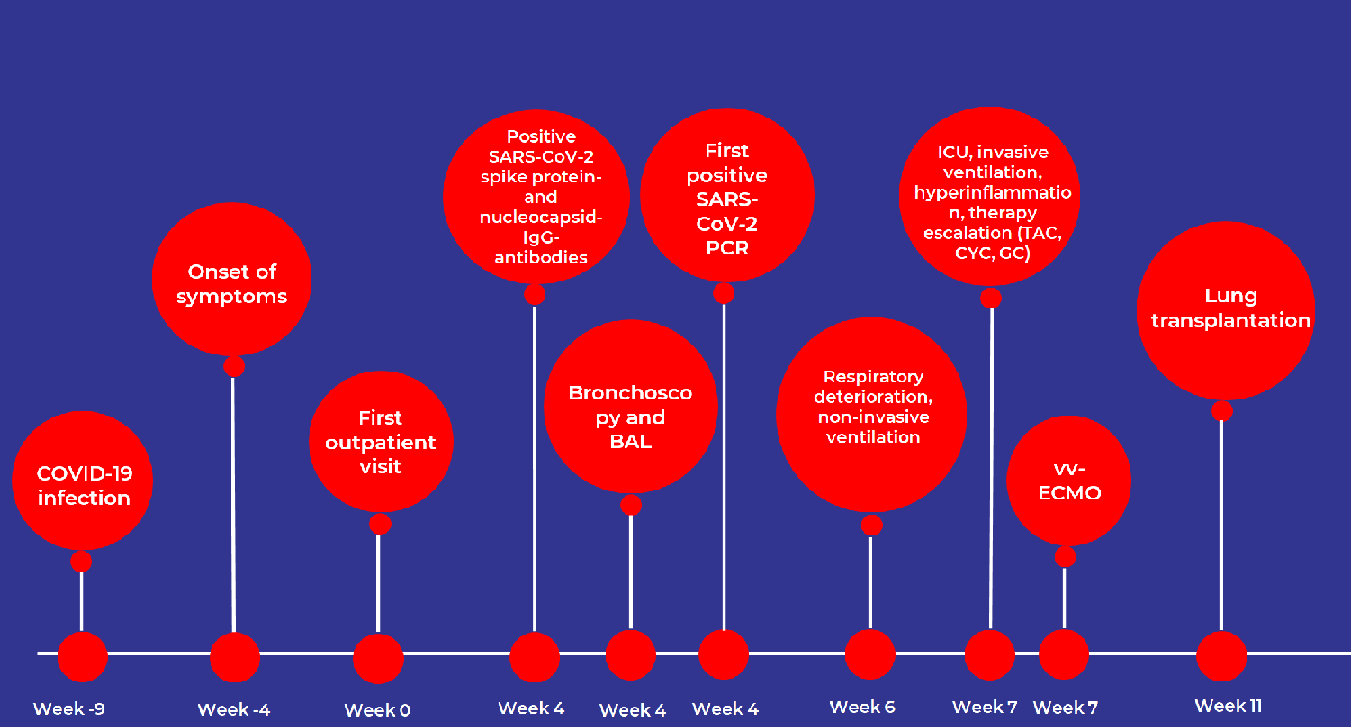


**Fig. S2:** Development of laboratory values during the inpatient and outpatient treatment: Creatine kinase (CK), C-reactive protein (CRP), Interleukin-6 and ferritin


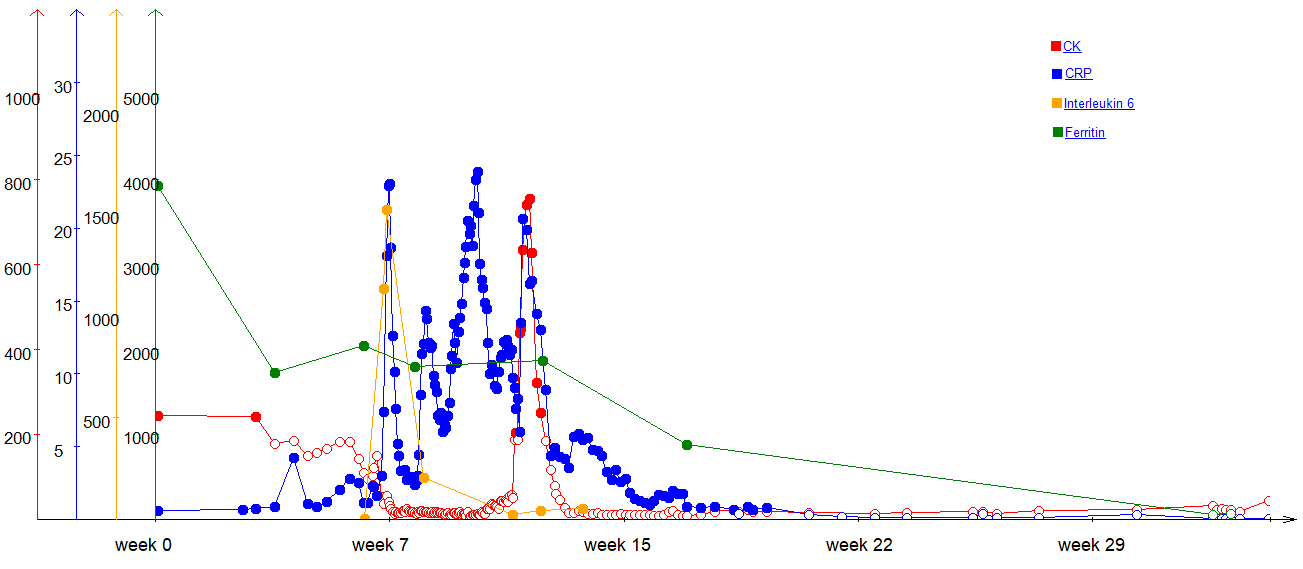


**Supplement 3:** List of the analysed genes in association with myositis:

ADAR, C4A, CAPN3, CCR1, ERAP1, FAS, FOXP3, HLA-B, HNRNPA2B1, IFIH1, IL10, IL12A, IL12A-AS1, IL23R, IRAK1, KLRC4, LAMA2, MEFV, PSMB4, PSMB9, PSTPIP1, RNASEH2A, RNASEH2B, RNASEH2C, SAMHD1, SPP1, STAT4, STING1, TLR4, TNFRSF1A, TREX1, UBAC2, NAB1, TYK2, YDJC, PTPN22, FAM167A, DGKQ
